# Costing for universal health coverage: insight into essential economic data from three provinces in Cambodia

**DOI:** 10.1186/s13561-019-0246-6

**Published:** 2019-10-30

**Authors:** Bart Jacobs, Kelvin Hui, Veasnakiry Lo, Michael Thiede, Bernd Appelt, Steffen Flessa

**Affiliations:** 1 Social Health Protection Programme, Deutsche Gesellschaft für Internationale Zusammenarbeit (GiZ), c/o NIPH, No.2, Street 289, Khan Toul Kork, P.O. Box 1238, Phnom Penh, Cambodia; 2 Social Health Protection Network P4H, Phnom Penh, Cambodia; 3 Department of Planning and Health Information, Ministry of Health, Phnom Penh, Cambodia; 4 Scenarium Group GmbH, Berlin, Germany; 5 Department of General Business Administration and Health Care Management, University of Greifswald, Greifswald, Germany

**Keywords:** Health service costing system, Feasibility, Step-down allocation, Unit cost, Health Centre, Hospital

## Abstract

**Background:**

Knowledge of the costs of health services improves health facility management and aids in health financing for universal health coverage. Because of resource requirements that are often not present in low- and middle-income countries, costing exercises are rare and infrequent. Here we report findings from the initial phase of establishing a routine costing system for health services implemented in three provinces in Cambodia.

**Methods:**

Data was collected for the 2016 financial year from 20 health centres (including four with beds) and five hospitals (three district hospitals and two provincial hospitals). The costs to the providers for health centres were calculated using step-down allocations for selected costing units, including preventive and curative services, delivery, and patient contact, while for hospitals this was complemented with bed-day and inpatient day per department. Costs were compared by type of facility and between provinces.

**Results:**

All required information was not readily available at health facilities and had to be recovered from various sources. Costs per outpatient consultation at health centres varied between provinces (from US$2.33 to US$4.89), as well as within provinces. Generally, costs were inversely correlated with the quantity of service output. Costs per contact were higher at health centres with beds than health centres without beds (US$4.59, compared to US$3.00). Conversely, costs for delivery were lower in health centres with beds (US$128.7, compared to US$413.7), mainly because of low performing health centres without beds. Costs per inpatient-day varied from US$27.61 to US$55.87 and were most expensive at the lowest level hospital.

**Conclusions:**

Establishing a routine health service costing system appears feasible if recording and accounting procedures are improved. Information on service costs by health facility level can provide useful information to optimise the use of available financial and human resources.

## Background

Awareness about the costs of health services is a prerequisite to delivering these services effectively and efficiently in the context of limited financial resources [1]. Knowledge of costs aids managers at health facilities and administrative entities to deliver optimal health care by facilitating accurate planning and budgeting, as well as efficient resource allocation. Social health protection schemes can use knowledge of health service costs to determine reimbursement rates and improve purchasing, thereby potentially improving the quality of care [2–6].

Despite the promising applications of costing results for management and health financing, the costs of health services are rarely defined in resource poor countries because of the unavailability of data at the health facility level, and the lack of personnel with the necessary skills to reliably conduct the assessments [2–4]. As a result, costing studies in these countries tend to be expensive, infrequently conducted by consultants, and limited to a few facilities and health services [7]. Constraints to the use of the costing results include a low tendency for evidence-based policymaking and limited familiarity with economic evaluation findings amongst decision-makers [8, 9]. Because of the challenges involved with and infrequency of cost-assessments in low- and middle-income countries, the focus tend to be on diseases rather than the health system [10].

The situation for Cambodia is similar as the number of studied health facilities is rather small, often limited to a specific type of health facility and with long time periods in between. In 2002, Fabricant analysed data from the 2001 financial year for four provincial hospitals, eight district/referral hospitals, two health centres with beds (HCBs), and 16 health centres without beds [11]. In 2009, using 2007 data, Collins et al. assessed the costs of service packages delivered by health centres and hospitals to support the Cambodian Ministry of Health (MOH) in costing its second Health Strategic Plan (2008–2015) [12, 13]. However, the models used for these normative costing exercises had many assumptions that were no supported by empirical evidence. Another study [14] analysed the service costs at 10 public hospitals at various levels for the years 2011–2012.

Other studies in Cambodia analysed the costs of specific health services or conditions such as dengue fever [15], cervical cancer [16], and childhood survival [17], or the costs of specialised service providers such as a trauma hospital [18]. Consequently, relevant, up-to-date information regarding the actual costs of public health services in Cambodia is not available, and there is a need to update such knowledge based on a standard methodology. In the context of health financing for universal health coverage, such information should be available at regular intervals from a representative sample of health facilities at various levels to aid in cost containment and quality improvement in purchasing by social health protection schemes [19].

Following three decades of civil conflict and the destruction of the country’s administrative and health systems, in 1995 Cambodia embarked on a series of health reforms organised around the concept of district health systems [20]. In this system, a health district (referred to as an operational district; OD) is established along population norms, with ODs encompassing 100,000 to 200,000 people, and often cuts across administrative district boundaries. Often, several ODs make up a province, and each OD has a hospital that delivers a complementary package of activities (CPA) at one of three levels; CPA1 to CPA3. CPA1 hospitals have 40–60 beds, provide no surgical services and have no blood bank, often because of their proximity to provincial or national hospitals. CPA2 hospitals (60–100 beds) provide surgical services, and CPA3 (100–250 beds) is reserved for provincial and national hospitals that have a wide range of specialised health services [20]. ODs also have health centres that serve about 10,000 to 20,000 people each, and deliver the minimum package of activities (MPA), comprising preventive health services and basic curative care. Former administrative district hospitals were transformed into HCBs, which provide rudimentary inpatient care in addition to MPA. In 2015, there were 25 CPA3 provincial hospitals (for 25 provinces), 68 CPA2 and CPA1 district hospitals (for 95 ODs), six national hospitals, and 1248 health centres [21].

Following the introduction of user fees in 1996, Cambodia implemented a series of health financing interventions aimed at increasing access to health services, primarily targeting impoverished households. These initiatives included community-based health insurance, health equity funds (HEFs), vouchers, internal and external contracting, and a midwifery incentive scheme [22–26]. From 2017, with the endorsement of the Social Protection Policy Framework [27], the social health protection system has included social health insurance for private formal sector employees (2017) and civil servants (2018). Prior to the introduction of these schemes formal private sector employees benefited from the Work Injury Insurance only [28]. Health equity funds provide coverage to about 2.7 million poor people. Together these schemes covered about 4.5 million people. Reliable and routinely updated information on unit costs of health services could thus greatly aid in strengthening strategic purchasing and potentially improve the quality and efficiency of health services.

Here we report on the results of the initial phase of the establishment of a routine health service costing system using a standard methodology in three provinces of Cambodia. We describe the process of collecting information in the Methods section to highlight the challenges that need to be addressed in establishing such a costing system. We further discuss how the findings can be used to improve technical and allocative efficiencies based on results presented in the respective sections.

## Methods

### Study setting

Health facilities were selected based on the three provinces where the Deutsche Gesellschaft für Internationale Zusammenarbeit (GIZ) Social Health Protection Programme operates to inform policy level regarding the feasibility to operationalise interventions: Kampot; Kampong Thom; and, Kep. For the first two provinces, the total number of selected public health facilities included 20 health centres (including four HCBs), two CPA2 district hospitals, and two CPA3 provincial hospitals. The number and kind of health facilities in each province was similar. For the third province, Kep, only the CPA1 hospital was selected. Table 1 provides more details about the provinces, while Table 2 elaborates selected variables of the HCBs and hospitals that were part of the study.

**Table 1:** Provincial characteristics

	Kampong Thom	Kampot	Kep
Land area^a^ (km^2^)	13,814	4873	336
Population^b^	690,414	611,557	38,701
Population density (people/km^2^)	50	125.5	115
City population^c^	61,348	60,851	19,573
Number of villages	739	488	18
Number of health centres	53	64	5
Total number of public hospital beds	194	303	13
Hospital beds per 1000 population	0.28	0.50	0.34
Physicians per 1000 population	0.09	0.10	0.72
Distance from Phnom Penh	199 km	152 km	167 km
Poverty rates^d^	29.1%	20.4%	16.5%

^a^https://en.wikipedia.org/wiki/Provinces_of_Cambodia; ^b^National Institute of Statistics. Cambodia Intercensal Population Survey 2013. 2013. Phnom Penh, Ministry of Planning; ^c^United Nations Population Fund. Urbanization and its linkage to socio-economic and environmental issues. 2014. Phnom Penh, UNFPA; ^d^Asian Development Bank. Cambodia: Country Poverty Analysis 2014. 2014. Manila, Asian Development Bank

**Table 2:** Health centres with beds and hospitals key features

	Health Centre with Beds				CPA1	CPA2		CPA3	
	Kampong Thom		Kampot		Kep	Kampong Thom	Kampot	Kampong Thom	Kampot
Beds	32	31	15	30	28	55	52	120	133
OPD*	10,852	14,396	3540	7184	8205	16,447	6446	8972	9805
IPD patients*	590	509	–	551	966	5690	2189	8145	10,110
IPD day	1382	1157	–	2075	5039	26,623	8123	35,686	44,229
Occupancy (%)	12	10	–	19	49	133	43	81	91
ALOS (days)	2.34	2.27	–	3.77	5.22	4.68	3.71	4.38	4.37

*ALOS* average length of stay, *IPD* inpatient, *OPD* outpatient consultations; * per annum

### Data collection

Data was collected from May to September 2017, and related to the 2016 financial year. Three manuals (one each for hospitals, health centres without beds, and HCBs) were prepared, with detailed instructions for the data collection and analysis. Each manual was accompanied by a data collection tool developed in Microsoft Excel (all available upon request from the authors). The data collection tool was organised by expenditure category: labour costs; stores (medicine, consumables and vaccines; laboratory supplies; domestic supplies; food; linen and clothing); transport and travel; medical equipment; vehicles; buildings; and, general expenditures (electricity; water; printing). For fixed assets such as buildings, medical equipment, and vehicles, information was also collected about the year of construction or purchase, the current degree of functionality, and associated costs for maintenance and repairs.

The data collection tool was also designed to accept income data. This included direct patient fees, income from various social health protection schemes such as HEFs, and health financing interventions such as vouchers or midwifery incentive schemes, government grants (cash, salaries, drugs and materials, allowances), and donations. Government grants also included depreciations, which were automatically calculated from the information entered in the expenditure sheet. Including both income and expenditures in the same sheet allowed for comparison and assessment of data quality, as the balance should have been zero unless the facility made a profit or a loss.

Additional sheets in the data collection tool related to: equipment (year of purchase, purchase price, department, condition, maintenance costs); buildings (year built, departments housed, initial price, floor size - in case there was no construction price, maintenance costs, main material -wood or cement); vehicles (year of purchase, maintenance, condition); medicine and materials (by department); personnel (department, position, salary and other income sources); and, basic statistics (outpatient consultations, inpatient admissions and inpatient days by department for facilities with beds, services provided by department, and support services such as laboratory tests or imaging).

Prior to collecting data, introductory workshops were held at each province by the Department of Planning and Health Information (DPHI) of the Cambodian Ministry of Health (MOH), with provincial health management officials, district administrators, and representatives of the target facilities. At the workshop, participants were introduced to the study objectives and data collection officers and informed of the types of data and documents required, and whether they should be in electronic or physical format. Data for costs was derived from various levels, depending on availability, and included the health facilities, respective ODs or provincial health departments (PHDs), and selected departments of MOH.

For remuneration of staff members, electronic salary records were received from PHDs, while overtime was obtained from ODs. When staff members worked at different departments they were asked to estimate their time spent at each department. Staff income from other sources, such as midwifery incentive schemes, was obtained from ODs for their respective health centres, and from financial reports for hospitals. Allocation of this revenue amongst staff members in health centres was obtained through interviews with the respective manager.

Consumption of drugs and medical materials supplied through the Central Medical Store was obtained from the OD pharmacy database for health centres, and from pharmacy managers at the hospitals. Information on drugs procured at pharmacies was obtained from the account booklet for health centres, and from invoices for hospitals. No details were available for drug consumption by department at health centres, and thus this information had to be derived from the drug registration books, where all prescriptions were chronologically recorded. This information was counted for two months that represent two distinct seasons (January and June) and extrapolated to the 12-month financial year. On the other hand, all hospitals except the CPA2 facility in Kampot used the web-based Central Medical Store Databank to record medicine and material consumption by department. The costs of drugs and materials were derived from the invoice documents provide by the Central Medical Store to each OD.

The costs and ages of vehicles were derived from inventory lists at ODs and PHDs, complemented by visual inspection of their functionality at the facility. An equipment list existed for most facilities but tended not to be updated after 2012. This list was thus updated by the data collection officers through visual inspection of departments. When original prices were not available, equivalent prices supplied by MOH were substituted. Only equipment with a minimum value of US$1000 was considered in the study, but lump sums were added for basic equipment in the range of US$1500-US$3000 for health centre departments, and US$5000- US$25,000 for hospital departments.

Information regarding age and price of buildings was obtained from inventory lists at ODs and PHDs, while the size was derived from construction plans. When plans were not available, or one building housed several departments, the size was determined through measurement.

General expenditures by facility were calculated from monthly health financing reports submitted to ODs and PHDs. Income from various sources such as the government, user fees, and payments by social health protection schemes also came from these reports.

Use of services from service centres, such as laboratory tests and imaging (sonogram, radiology), had to be retrieved from the logbooks, with a sample of 1–2 months used to enable allocation by department. Only one provincial hospital (in Kampot) was using the electronic Laboratory Information System, which enabled the allocation of tests per department for the entire year. Data on service delivery and uptake by health facility came from Health Information System reports provided by DPHI.

When possible, electronic records in Microsoft Excel were retrieved and used. Prices of equipment and useful life expectancy were obtained from the Hospital Department at MOH. Data was collected over six weeks by two teams of trained researchers and was conducted simultaneously with data entry and analysis from May to September 2017.

### Costing method

In this study, cost is defined as the financial expression of the consumption of resources expressed in currency units (US$). The provider perspective was applied, whereby only provider costs are considered, unlike other cost concepts such as intangible costs or household costs [29]. The calculation of provider costs follows a standard step-down allocation methodology, which is frequently applied in the costing of health services [1, 4].

Full costs are calculated as much as possible, representing the total reduction in the value of resources of a health care provider within one year, and computed irrespective of the year and source of payment. All costs were allocated to different cost centres according to where the respective resources were consumed.

The step-down allocation strictly distinguishes between direct costs (occurring only because a specific service is rendered), and indirect costs or overheads (occurring for the general operation of the unit). Direct costs are allocated to the final costing units while indirect costs are allocated to the cost centres where they occur. The costs of service centres are allocated, stepwise, to the final cost centres. Finally, the total costs of the cost centres are allocated to the respective cost unit.

For hospitals, service centres included administration, laundry, kitchen, pharmacy, laboratory and imaging, while final cost centres were the departments of outpatient consultation, general medicine, paediatrics, maternity, other inpatient departments, and other services.

Cost centres for health centres were administration, outpatient consultations (OPD; encompassing all curative services except for chronic patients), maternity (delivery), services for chronic patients (patients requiring three or more contacts for their condition), and preventive services (vaccinations, antenatal care, and family planning).

Costing units for hospitals were: cost per OPD visit, patient, inpatient-day, and bed-day for the entire hospital; cost per patient and cost per bed-day for each of the inpatient cost centres (surgery, general medicine, paediatrics, maternity, and other inpatient departments); and cost per patient for other services (e.g., HIV counselling). For health centres, the costing units were cost per curative service (including minor surgery), delivery, contact per patient with chronic disease, preventive services, and other services.

### Additional analysis

This costing study aims to produce results based on a standard methodology to enable comparison with other similar studies. As several other studies did not include depreciation charges into their analysis, results are presented with and without depreciation. Straight line depreciation was used, whereby the value of the item was divided by its useful lifespan.

To enable a comparison of service costs in relation to quality of care, we mapped the costs of OPD visits with the quality of care score for each respective health centre. As a proxy for quality of care, we used outcomes from the Level II Quality Assessment of Health Care Facilities [30]. This assessment has been conducted annually since 2010 at all public health facilities within the country by MOH to assess quality of care. It includes determinants of structural quality (e.g., staffing patterns, buildings, equipment, and availability of electricity), process quality (e.g., documentation), and technical quality (e.g., infection rates, routine clinical procedures, and behaviour of staff towards patients).

Results are provided per province for all facility types. For an overview of costs by OPD and IPD, data were merged by facility level, irrespective of location. To assess the variability in costs for selected health services across the lowest level facilities using a standard deviation and variation coefficient, health centres and HCBs were merged together.

To compare costs from other studies conducted in Cambodia, we converted all values to 2016 values using annual inflation rate figures.

## Results

### Provincial features

Kampong Thom had the largest provincial population, with about 690,000 people, compared with 612,000 in Kampot and 39,000 in Kep. Kampong Thom was also the largest geographic province in the study, with a size of 13,814km^2^, compared to 4873km^2^ for Kampot. It also had the lowest population density, with only 50 people/km^2^; 2.5 times less than Kampot (Table 1). The number of villages in Kampong Thom was 739; 51% more than in Kampot. Kampong Thom had the highest poverty incidence, 29.1%, but the lowest number of physicians and hospital beds per 1000 population of the three provinces.

The workload of the facilities differed considerably (Table 2). In terms of admissions, CPA3 hospitals had the biggest workload, followed by CPA2 hospitals. For the latter, the number of inpatient admissions (IPD) ranged from 2189 (Kampot) to 5690 per year (Kampong Thom). The CPA1 hospital’s performance was similar to the performance of the HCBs. However, bed occupancy rate and the average length of stay (ALOS) were quite different between facilities. The occupancy rates were very low for the CPA2 hospital in Kampot (43%), the CPA1 hospital (49%), and the HCBs (0–19%). One HCB (15 beds) had no admissions during the study period. ALOS is also rather low for each level of care, suggesting that the complexity of services offered at the institutions is also relatively low.

### Total cost and unit cost

#### Health centres

Table 3 provides the actual annual costs by type of health centre and province. For all health centres, including HCBs, salaries and wages made up the largest proportion of costs (44–50%), followed by medicine and materials (37–44%).

**Table 3:** Annual costs and costs per service unit for health centres and health centres with beds (US$)

	Health centres			Health centres with beds		
	Kampong Thom	Kampot	Average	Kampong Thom	Kampot	Average
	Mean (% of total)					
Salaries and wages	26,208 (45)	31,260 (44)	28,644 (44)	42,326 (46)	40,874 (54)	42,100 (50)
Stores	22,729 (39)	33,565 (47)	28,147 (44)	33,720 (37)	28,402 (37)	31,061 (37)
Other	8905 (15)	6380 (9)	7642 (12)	14,254 (16)	7058 (9)	10,656 (13)
Total cost per year	57,662	71,206	64,434	91,300	76,334	83,817
	Mean (standard deviation)					
Contacts per year	22,989 (8721)	23,633 (7344)	23,096 (7795)	16,750 (4148)	27,720 (417)	22,235 (6775)
Cost/contact	2.78 (0.88)	3.21 (0.82)	3.00 (0.85)	5.37 (0.70)	3.81 (1.03)	4.59 (1.15)
OPD per year	16,405 (6143)	4312 (1993)	10,359 (7646)	12,624 (2506)	5362 (2577)	8993 (4678)
Cost/OPD	2.33 (1.10)	4.89 (1.36)	3.61 (1.78)	4.04 (1.28)	5.88 (1.44)	4.96 (1.54)
Deliveries per year	125 (101)	181 (115)	153 (109)	169 (81)	280 (100)	224 (98)
Cost/delivery	662.52 (1250)	129.39 (31.17)	413.72 (926)	98.91 (43.46)	158.56 (14.97)	128.74 (43.48)

Total annual costs per health centre were lowest in Kampong Thom (19% less than in Kampot), while contacts per year were similar; thus, the cost per contact was also lowest in Kampong Thom (US$2.78, compared to US$3.21 for Kampot). In Kampot, these contacts were mainly for preventive services, and the annual number of OPD visits was only about a quarter of the number of OPD visits at Kampong Thom health centres. Consequently, the cost of an OPD visit in Kampong Thom was US$2.33; less than half the cost in Kampot (US$4.89).

On the other hand, the annual number of deliveries was lowest in Kampong Thom, partly due to the fact that some health centres had very few deliveries (ranging from 2 to 350). As such, the average costs of delivery were much higher in Kampong Thom than in Kampot. For example, one health centre had only two deliveries, which resulted in an average cost per delivery of US$3673.5 in that health centre. If the three facilities with the lowest delivery frequencies (2, 10, and 24, respectively) are excluded, the cost per delivery of the remaining institutions is an average of US$106.6.

There is considerable variation in the costs per service unit between health centres and provinces. Table 4 shows that the average costs per visit, per OPD, and per treatment of a chronic patient at a health centre are significantly lower in Kampong Thom than in Kampot. The costs per delivery in Kampong Thom exclude the three health centres with few institutional deliveries. After reallocating these costs to the OPD department and ignoring maternity in these three health centres, the costs per OPD attendance and per delivery are lower in Kampong Thom than in Kampot. There is no explanation for this cost difference, which warrants further research.

**Table 4:** Average cost of services at health centres and their standard deviation and variation coefficient (merged results for health centres and health centres with beds)

	All health centres			Kampong Thom			Kampot		
	Av	SD	VC	Av	SD	VC	Av	SD	VC
Visit	3.24	0.96	0.30	3.16	1.06	0.33	3.33	0.80	0.24
Outpatient consultation	3.88	1.79	0.46	2.67	1.21	0.45	5.09	1.29	0.25
Delivery	107.29	43.72	0.41	70.55	25.32	0.36	135.87	30.34	0.22
Chronic	40.95	35.99	0.88	28.73	22.28	0.78	53.17	40.25	0.76

*Av* average cost, *SD* standard deviation, *VC* variation coefficient

Even within each province, the variation of costs per service unit is high (Table 4). The variation coefficient (calculated as the standard deviation divided by the mean value), which measures the proportion of results that is equal to the mean costs, is 33% of the costs per visit in Kampong Thom and 24% in Kampot. For OPD visits, it is 45% and 25%, respectively. Thus, there is a higher variability in costs across health centres in Kampot (range US$2.86–6.90) than in Kampong Thom (range US$1.34–5.01).

#### Hospitals

As expected, the cost per hospital bed strongly depended on the level of care, as reflected in Kampot where the annual costs per bed were US$13,215 at the CPA3 hospital and US$6038 at the CPA2 hospital. Curiously, in Kampong Thom hospitals the costs were US$17,321 and US$17,644 in the CPA3 and CPA2 hospitals, respectively. For the CPA1 hospital in Kep, the costs per bed were US$13,154, which is equal to the costs per bed of the highest level hospital in Kampot. However, it seems that the number of official beds and the real number of beds did not always match.

Table 5 shows the costs per service unit in the hospitals. The costs per service unit do not necessarily correlate with the level of care; it appears that the workload (occupancy rate) seems to be a better determinant of cost than the level of care. As such, the CPA1 hospital had extraordinarily high unit costs. Since the CPA2 hospital in Kampong Thom had a higher workload than its equivalent in Kampot, unit costs for services were lower, except for tuberculosis. The contrary was observed for CPA3 hospitals, whereby costs in Kampot were lower than in Kampong Thom due to a higher workload.

**Table 5:** Cost in hospitals (US$)

	CPA1	CPA2		CPA3	
		Kampong Thom	Kampot	Kampong Thom	Kampot
Total cost					
Salaries and wages	101,431.82	283,663.02	197,053.53	680,464.25	681,167.29
Stores	171,140.15	338,205.20	46,156.33	1,044,420.50	795,492.27
Other	95,734.23	154,482.30	70,763.76	353,574.85	281,019.58
Total cost per year	368,306.20	776,350.52	313,973.62	2,078,459.60	1,757,679.14
Cost per service unit					
OPD	9.65	2.29	9.44	49.76	33.31
IPD patient	291.45	61.45	111.61	178.88	137.54
IPD day	55.87	25.14	30.08	44.97	31.44
Surgery	–	24.71	25.03	39.79	19.79
General Medicine	40.39	30.49	21.24	32.21	35.86
Paediatrics	40.07	16.80	62.90	47.03	33.63
Maternity	66.72	19.11	36.38	60.89	32.24
TB	–	209.11	27.88	46.36	22.79
Other Inpatient	–	19.89	–	51.54	21.54
Other Services	252.56	–	–	–	–

Higher level hospitals did not necessarily consume more resources to produce one service unit than lower level hospitals, as the CPA1 facility had the highest unit costs for all cost centres except OPD and paediatric admissions.

#### Costs without depreciation

Table 6 provides depreciation charges for equipment, vehicles and buildings by type of facility and location. The costs per service unit declined by an average of 7% (ranging from 3% to 26%) when disregarding depreciation charges. This was 12% (range: 5–22%) for hospitals, 10% (range: 3–26%) for HCBs, and 5% (range: 3–10%) for health centres. As the costs of some buildings and equipment were already written off, these figures tended to underestimate the necessary capital input requirements to maintain the facilities at their existing levels.

**Table 6:** Depreciation by type of facility and province (US$)

	Health centre		HCB		CPA1	CPA2		CPA3	
	KThom	Kampot	KThom	Kampot		KThom	Kampot	KThom	Kampot
Equipment	1458	1463	2103	1765	23,513	29,553	42,525	85,704	127,270
Vehicles	400	183	3056	488	30,474	9884	10,344	19,483	15,594
Buildings	1003	1930	3932	2000	27,178	6759	6150	5930	20,120
Total Depreciation	2861	3575	9091	4252	81,165	46,196	59,019	111,117	162,984
Per service unit	0.16	0.17	0.49	0.15	12.57	1.64	5.82	2.44	2.98

*KThom* Kampong Thom

**Table 7:** Average cost of OPD consultation and IPD admission by level of facility (in US$)

	Health Centre	HCB	CPAI	CPAII	CPAIII
Outpatient	3.00	4.96	9.65	5.87	41.53
Inpatient day		4.46	55.87	27.61	38.21

The relevance of depreciation also depended on the cost centre. Departments with a high reliance on equipment and buildings had a stronger reduction in unit costs if depreciation was not considered. For instance, the average costs of the CPA3 hospital in Kampong Thom decreased by 5% if depreciation was not included. Without considering depreciation, the average costs decreased by 8% for OPD, 10% for surgical, 5% for general medicine, and 3% for paediatrics and maternity departments.

#### Average costs by type of facility

Table 8 provides an overview of the average costs for OPD consultations and IPD admissions by health facility level. Costs increased by the complexity of services, although the CPA1 hospital was an exception. Its OPD consultation costs were more than OPD costs in CPA2 hospitals, and the costs of an IPD-day in the CPA1 hospital were twice the costs of the same service in CPA2 hospitals and 46% higher than an IPD-day in CPA3 hospitals.

**Table 8:** Income in US$ (% of total)

	Health centre		Health Centre w Beds		CPA1	CPA2		CPA3	
	Kampong Thom	Kampot	Kampong Thom	Kampot		Kampong Thom	Kampot	Kampong Thom	Kampot
Patient fees	6999 (12)	7086 (10)	12,408 (13)	10,717 (10)	28,344 (7)	220,933 (27)	34,065 (11)	452,231 (22)	474,234 (25)
Government	51,636 (88)	65,190 (90)	82,270 (87)	99,586 (90)	362,848 (93)	599,885 (73)	279,905 (89)	1,632,234 (78)	1,410,283 (75)
Other	–	2 (0)	–	–	–	–	–	–	
Total	58,634	72,278	94,678	110,303	391,192	820,818	313,970	2,084,465	1,884,517
Patient fees									
User fees	37%	70%	44%	60%	73%	63%	79%	42%	71%
HEF	37%	10%	26%	13%	27%	23%	21%	36%	14%
NSSF	0%	1%	0%	26%	0%	0%	0%	0%	0%
Other	26%	19%	30%	0%	0%	14%	0%	22%	15%
Government income									
In Cash	5%	3%	4%	2%	7%	11%	0%	11%	4%
Salaries and Wages	39%	33%	43%	42%	24%	24%	62%	24%	24%
Drugs and Materials	41%	50%	38%	41%	46%	54%	15%	58%	51%
Depreciation	5%	5%	10%	4%	22%	6%	21%	6%	12%
Midwife Incentive Scheme	4%	9%	4%	11%	1%	3%	2%	1%	6%
Other Government Grants	6%	0%	1%	0%	0%	2%	0%	0%	4%

*HEF* health equity fund, *NSSF* national social security fund

### Actual income

Table 8 shows the income of health care facilities by type and location. The Government of Cambodia was the main funder, but its proportion of funding depended on the level of the health provider. On average, 88% of the income of all institutions came from the government. As seen earlier, the largest share of these contributions was for staff, medicine and medical materials. Salaries and wages made up 36% of total government contributions (range: 24–62%). The average was 32% in hospitals, 43% in HCBs, and 36% in health centres. Medicine and medical materials amounted to 44% of all government-provided income, ranging from 15% to 60%: 45% in hospitals (range: 15–58%), 45% in health centres without beds (range 29–60%), and 40% in HCBs (range: 32–48%). The proportions of cash (4%), compensation of depreciation (7%), and midwife incentive schemes (6%) were low in comparison.

Generally, the higher the facility level, the greater the relevance of user fee revenue. The income from patient user fees varied substantially between institutions but was an average of 12% overall. For hospitals, such income was 18% (range: 7–27%); for HCBs this was 11% (range: 9–16%); and for health centres it was also 11% (range: 4–22%). Out-of-pocket expenses among patients made up the largest proportion of user fees, accounting for an average of 58% of all user fees. Out-of-pocket expenses accounted for 66% of user fees in hospitals, 54% of user fees in HCBs, and 57% of user fees in health centres. Health equity funds contributed 24% to the average amount of user fees. Social health insurance contributions by the National Social Security Fund were not relevant in 2016; however, other insurance schemes, such as community-based health insurance, made up 11% of all fees. For some health centres this source of income was quite significant, constituting up to 50% of fee income.

### Efficiency, based on quality of care and costs

**Fig. 1:**
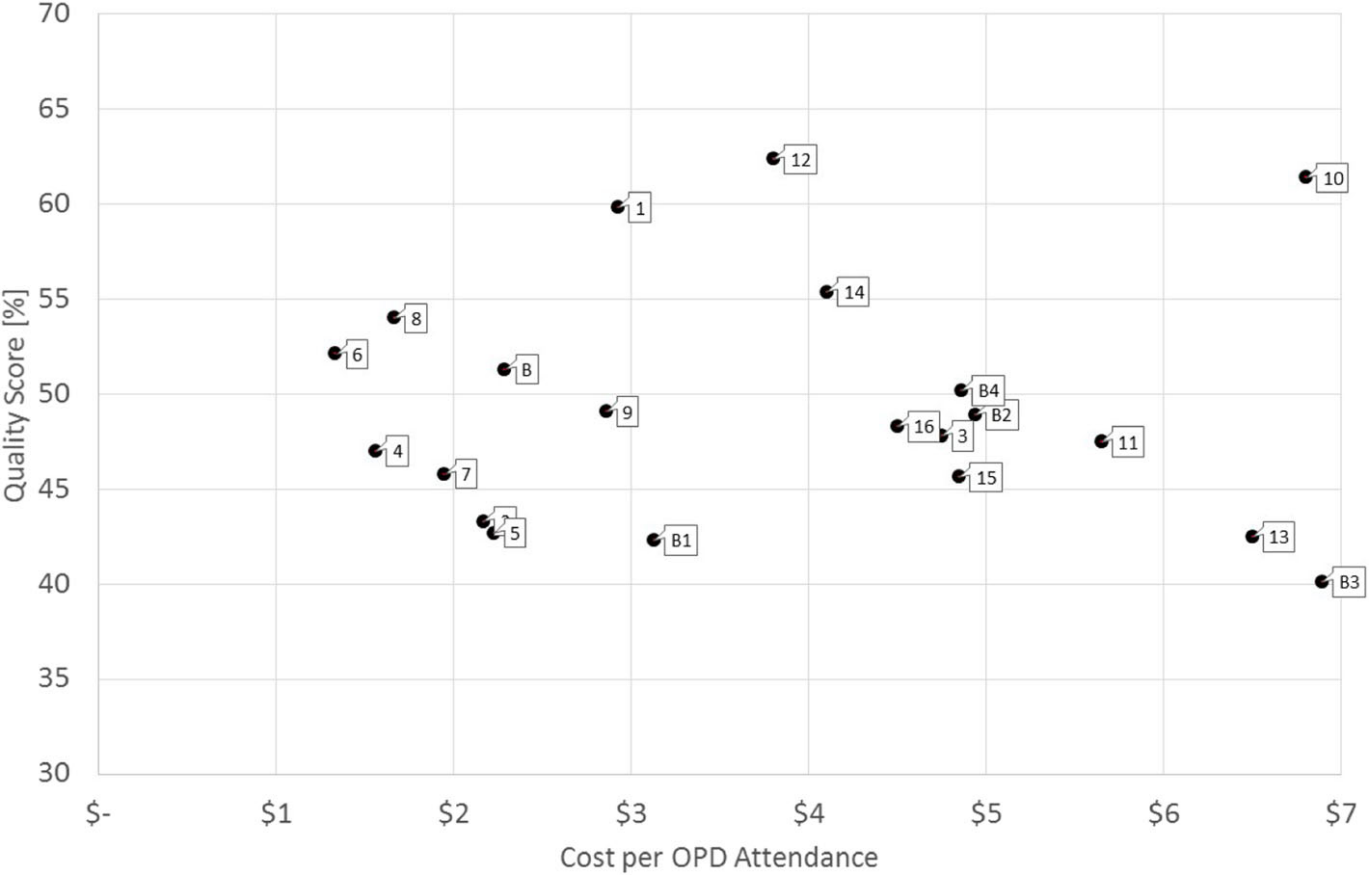
Efficiency diagram of health centre costs and quality scores (per OPD attendance)

Figure 1 plots the costs of an OPD consultation at a health centre against its quality score. Facility 12 achieved the best quality score (62) at a cost of less than US$4.00, while the cost of treatment at facility 10 was about US$6.80 for a similar level of quality. Facility B3 had the lowest quality of care (40%), but had the same cost requirements for an OPD treatment as facility 10.

### Time trend of costs

Table 9 compares the various health service costing studies conducted to date in Cambodia, with monetary values adjusted to 2016 values. This enabled the comparison of prices over time and suggests that medical inflation outpaced price inflation at all facility types. The one exception was the cost per bed-day at HCBs, where a marked decrease in costs was observed from 2011 to 2016. Table 10 compares the unit costs for an inpatient day and an outpatient consultation with results from neighbouring countries in 2016 US$ values.

**Table 9:** Health service costs in Cambodia (US$ adjusted to 2016 values)

	Fabricant [11]	Collins [12, 13]	Martin [14]	Current study
Kind of facility	2001*	2007*	2011*	2016*
Per bed day				
CPA1 hospital	9.04	15.51	12.81–17.08	55.87
CPA2 hospital	10.42	20.61		27.61
CPA3 hospital			18.15–26.69	38.21
HCB	3.47		25.62–30.96	4.46
Per OPD consultation				
CPA1 hospital			8.54–16.01	9.65
CPA2 hospital			5.34–14.94	5.87
CPA3 hospital			13.88–29.89	41.53
Health centre	1.83	2.46		3.00
HCB				4.96

*HCB* health centre with beds; * year of study

**Table 10:** Health service unit costs in neighbouring countries (US$ adjusted to 2016 values)

Country	Year of data	Cost per inpatient-day	Cost per outpatient consultation
Cambodia	2016	Primary hospital: 41.74^a^ Secondary hospital: 38.21	Primary hospital: 7.76^a^ Secondary hospital: 41.53 Health centre: 3.00
Lao PDR*	2005	Primary hospital: 25.07 Secondary hospital: 32.70 Tertiary hospital: 44.66	Primary hospital: 6.80 Secondary hospital: 10.07 Tertiary hospital: 14.91 Health centre: 9.96
Thailand*	2017	Primary hospital: 75.84 Secondary hospital: 98.95 Tertiary hospital: 135.15	Primary hospital: 19.25 Secondary hospital: 27.30 Tertiary hospital: 40.38 Health centre: 14.88
Vietnam*	2005	Primary hospital: 37.47 Secondary hospital: 48.88 Tertiary hospital: 65.51	Primary hospital: 11.37 Secondary hospital: 16.13 Tertiary hospital: 23.87 Health centre: 11.65

*source: World Health Organization. *CHOosing Interventions that are Cost-Effective*. 2017; http://www.who.int/choice/cost-effectiveness/en/; ^a^unweighted average CPA1 and CPA2 hospitals

## Discussion

Significant effort was required to gather data from 25 public health facilities in three provinces – 20% of which were hospitals of various levels. None of the target facilities had all required information available at the facility level. Only some facilities were able to provide data in electronic form, or only for selected services, cost categories or departments, and often data had to be copied from physical registers into electronic form for analysis. The three provinces varied in size, population density and poverty rate. The services provided by the respective health facilities also varied in terms of quantity, quality and costs.

While Kampot province had the highest population density, its health centres had only a quarter of the OPD consultations of health centres in Kampong Thom. This could be because of a failing referral system, whereby patients bypassed the health centre and sought care directly at hospitals. However, the number of OPD visits at the CPA2 hospital in Kampot was much lower than its equivalent in Kampong Thom, and only about 9% higher for the CPA3 hospital, suggesting that patients primarily sought care at private health facilities; a practice observed elsewhere in Cambodia [31]. While the dominance of the private sector may have ramifications for out-of-pocket expenses for patients seeking care at such facilities [32, 33], it also increases the unit costs for public providers as it lowers the volume of health services they deliver; costs for an OPD in Kampot were more than double the costs in Kampong Thom (US$4.89, compared to US$2.33). This difference was maintained, albeit to a lesser extent, in HCBs as well.

Such differences were not observed in the costs per contact at health centres, largely because health centres in Kampot delivered more services (mainly preventive), than health centres in Kampong Thom. This is in line with previous findings indicating that the Cambodian public health sector performs well for preventive care [34], as such services are rarely delivered by the private sector because of insufficient financial incentives [35]. Still, the costs per contact were higher in Kampot than in Kampong Thom (US$3.21 and US$2.78, respectively), suggesting that fixed costs were higher for health centres in the former province than the latter. This is supported by the fact that health centres in Kampong Thom received US$57,622 per annum in 2016, compared to US$71,206 for health centres in Kampot. Several authors have highlighted the fact that service volume lowers unit costs [3, 5, 7].

The requirement to increase the quantity of health services to lower the costs of providing them was also exemplified by deliveries, for which Kampot outperformed Kampong Thom. Delivery costs were an average of US$662.50 in Kampong Thom health centres, compared to US$129.39 in Kampot. This was largely due to low performing health centres in Kampong Thom. The inverse was observed for HCBs, whereby the cost of a delivery in Kampong Thom was a third cheaper than in Kampot (US$98.91 and US$158.56, respectively). This occurred despite a lower volume of annual deliveries (169 in Kampong Thom, compared to 280 in Kampot) and higher average annual costs to run the facilities (US$91,300, compared to US$76,334 in Kampot).

Within the same province, the costs for delivering health services at health centres (with or without beds) varied widely, as indicated by the variation coefficient. This was especially true for Kampong Thom. This higher variation can be attributed to differences in utilisation; due to the lower population density and greater number of villages, there was likely a considerable difference in the volume of services delivered by health centres in Kampong Thom. Kampong Thom also had a higher poverty incidence than Kampot. Poor people tend to use public health centres more than the non-poor [36], and they may not be homogenously spread across the province. In that respect, judging efficiencies on the basis of average costs per service or contact alone may not be equitable, as poor people tend to reside in remote areas which are sparsely populated and where it is challenging to post and retain staff members [37, 38]. Hafidz et al. [39] found in Indonesia that the most efficient health facilities were located in areas with easy access and high population density. Thus, there is a need to consider such contextual factors.

The higher costs of HCBs in comparison to health centres without beds – 53% more per contact and 63% more per OPD consultation – coupled with the low bed occupancy rates (0–19%)- challenge the economic rationale for sustaining such facilities. Instead, it may be worthwhile considering alternative investments in ambulance services or other vehicles to facilitate the efficient transportation of patients to higher level health facilities, especially in areas with accommodating road infrastructure [40]. However, costing health services for a small number of health facilities makes generalisations challenging [7], and there is a need to further investigate the use of HCBs.

Such an assessment is also justified in the context of the country’s decentralisation and deconcentration reforms, which may consider delegating authority from existing ODs to smaller administrative districts [21, 41]. This may lead to an increase in public health facilities with beds, as district administrative officials may want to consider adding beds to facilities in their districts, potentially leading to the types of inefficiencies reported in Indonesia [42]. Conversely, this decision may improve the structural quality of hospitals and utilisation of services, as observed in India [43]. While noting that these types of facilities are considered efficient when bed occupancy rates are more than 80% [3, 39], the low rates observed at HCBs and the CPA1 hospital in this study suggest the need for caution in expanding or maintaining these types of health facilities.

The increase in the average costs for OPD and IPD visit at each health facility level indicates the importance of respecting a referral system. Costs per OPD consultation were highest at CPA3 hospitals (US$41.53) in comparison with lower level health facilities, and cheapest at health centres (US$3.00). To improve or sustain the referral system, considerable investments will be required to improve the quality of care provided by health centres [44–47]. Use of the Level II Quality Assessment Tool or its successor, the Quality Enhancement Tool, could improve the quality of care and thus the likelihood that more patients will consult health centres first when they become sick, especially when accompanied by other interventions [48–50].

The average cost per inpatient-day was also higher at CPA3 hospitals (US$38.21) than at other levels of health facilities. The figure for the CPA1 hospital (US$55.87) necessitates enlarging the sample size, but this high cost can be explained by the low bed occupancy rate (49%). Depreciation also made up a considerable proportion of the total costs for running this facility (22%), but only accounted for 6%–12% of total costs for CPA3 hospitals. Excluding depreciation costs would lower the costs per service unit for the CPA1 hospital by US$12.57, but this would still be higher than the costs per inpatient-day and per IPD patient at CPA3 hospitals.

All health facilities were largely dependent on the government for their operations budget. This was especially true for health centres, as these facilities primarily delivered preventive health services and charged low user fees for curative health services. At hospitals the income from user fees tended to be higher (around 22–27% of facility income), but this correlated with the number of health services provided, as shown by the CPA2 hospital in Kampot which had low service provision and low user fee revenue. Apart from charging higher fees, hospitals also benefitted from the availability of advanced medical equipment and support services, for which they could charge additional fees. However, in these cases caution should be exerted to avoid or reduce provider-induced demand for services. The proportion of user fees coming from HEFs correlated with the incidence of poverty in the province, although it was low for both the health centres (10–13%) and the provincial hospital in Kampot (14%). This contrasted with Kampong Thom, where the proportion of user fees from HEFs were 26–37% for health centres and 36% for the provincial hospital, which was more aligned with earlier reports on revenue derived from HEFs [51].

The largest share of government contributions to health facilities consisted of medicine and medical materials. Similar to our findings, drugs and supplies constituted the highest proportion of costs of a 200-bed hospital in Myanmar, while in Bangladesh it constituted more than half of the unit costs for service delivery [5, 52]. On the other hand, at Indonesian hospitals and Indian primary and tertiary health facilities, personnel constituted the largest cost component [3, 39, 53]. These differences may be due to the costs of drugs and materials and staff remuneration being below the market rate for the Cambodian public sector [54].

Despite the low staff remuneration costs, overall costs appeared to rise faster than the inflation rate (Table 9). This was especially true for the costs per bed-day, while the cost increase over time in constant US$ was only considerable for OPD consultations at health centres and the CPA3 hospitals. While these figures may suggest medical inflation, the observed increase may also be due to methodological differences with earlier studies, or the relatively small sample of facilities considered (especially for hospitals). Costs may have also escalated because of new technologies, provider-induced demand, changes in provider payment methods, more expensive and/or inappropriately prescribed medicine, lower degree of service delivery, changes in patient case mix, and the employment of more staff [55]. Such eventual cost escalations can best be monitored by having a routine health service costing system in place. A routine costing system, coupled to the results of the Quality Enhancement Assessment, would allow managers at the district, province, or national level to monitor progress at the facility level.

The findings from this costing exercise suggest the feasibility of establishing a routine health service costing system. However, similar to findings from other countries with resource constraints [4, 56], collecting the required data involved considerable energy, innovation, and patience. Such circumstances hint that establishing a routine health service costing system may not be feasible without initial improvements in recording systems and inventory methods.

Initially, a routine health service costing system could be established at a subset of public health facilities, comprising a nationally representative sample, which would provide data on a routine basis. The use of simple software programmes could ensure the accuracy of the information and enable convenient data extraction. This should not be an issue for hospitals but can be challenging for health centres, as 32% of health centres had no computer in 2017 [57]. Support services also pose a challenge to data collection, although software programmes such as the Laboratory Information System, currently implemented at only six hospitals, would aid in appropriately determining consumption by department. In the absence of appropriate computer programmes, registration procedures for support services could be adjusted to elicit consumption by department. Given the prominence of drugs and consumables as part of facilities’ annual income and service costs, related information should be made more accessible and usable. Information related to equipment should be updated on an annual basis and verified by the Hospital Department of the MOH.

To ensure validity and reliability, data should be verified at various levels, with data analysis done at the central level, considering available human resources. For example, information entered at the health facility could be checked by the respective OD, whereas data provided by the OD could be cross-checked by the PHD before forwarding to MOH. This upward cascade of information and verification processes should be feasible, as by 2017 all hospitals had web-based submission of health information system reports. However, this was only the case for 47% of health centres [57]. The sample of health centres to be included in a regular reporting system could be selected from among all health centres with web-based reporting, but this may bias the information collected, as facilities without computers and/or internet may be located in poor areas or ODs with suboptimal performance.

The wide cost variations observed in this study, however, call for a larger sample of health facilities to increase the reliability of final unit cost data if results are to be used for strategic purchasing purposes. This reinforces the need for careful selection of health facilities to be included in the national-representative final sample. Consideration should be given to facilities’ health service volume as well as contextual factors such as poverty incidence, population density and accessibility, all factors potentially affecting unit costs.

To promote the use of the data for decision-making, policymakers at all levels of the health system should be familiarised with the principles of economic evaluations [58], the arrangements for and use of evidence-based policymaking should be strengthened [59] while contextual factors such as the degree of decision-making by managers below national level and reliable budget allocation should be addressed [60]. Providing lower-level managers with more cash instead of in-kind provisions could also stimulate the use of the costing data.

### Limitations

Costing data and costing studies must be interpreted with care. Frequently, accounting and medical recording, as well as other documentation procedures, are not entirely reliable. Consequently, data for a health facility is not available or has to be collected from various sources, which may challenge its reliability. Furthermore, if the data is not produced automatically within an electronic routine reporting system, it may be of poor quality. The small sample size of this study does not allow generalisation of the findings. For many health facilities, and especially health centres, we calculated drug consumption and use of support services by department for only two months and extrapolated this to the entire year. For equipment, we also limited inclusion of equipment to items costing at least US$1000. Thus, these costs may be an underestimation.

## Conclusion

The establishment of a routine health service costing system among a nationally representative sample of public health facilities appears feasible in Cambodia, provided that recording, stock-keeping and accounting procedures improve. Variations in costs per service and patient contact were observed between similar health facilities and within provinces, and costs increased by health facility level, suggesting the need to reinforce the referral system. In one province there appears a need to carefully monitor the private health sector and stimulate the use of health centres for curative care. The costs for services delivered at HCBs, together with their very low bed-occupancy rates, calls for careful consideration of their viability, and their economic reality should be weighed against the political feasibility of modifying these centres.

## Data Availability

Data sheets in Microsoft Excel will be uploaded as requested.

## Abbreviations

ALOS: Average length of stay
CPA: Complementary package of activities
DPHI: Department of Planning and Health Information
GIZ: Deutsche Gesellschaft für Internationale Zusammenarbeit GmbH
HCB: Health centre with beds
HEF: Health equity fund
IPD: Inpatient admissions
MOH: Ministry of Health
MPA: Minimum package of activities
OD: Operational district
OPD: Outpatient consultation
PHD: Provincial health department
